# Maternal sleep quality during pregnancy and neonatal outcomes: A study from Riyadh, Saudi Arabia

**DOI:** 10.1371/journal.pone.0345007

**Published:** 2026-04-10

**Authors:** Wjdan A. Almutairi, Waleed M. Alshehri, Bader M. Almutairy, Thurayya Eid, Ashwaq A. Almutairi, Abdulaziz M. Alodhailah

**Affiliations:** 1 College of Nursing, King Saud Bin Abdulaziz University for Health Sciences (KSAU-HS), Riyadh, Saudi Arabia; 2 King Abdullah International Medical Research Center, Riyadh, Saudi Arabia; 3 Ministry of National Guard Health Affairs, Riyadh, Saudi Arabia; 4 Department of Medical-Surgical Nursing, College of Nursing, King Saud University, Riyadh, Saudi Arabia; 5 Department of Community and Psychiatric Mental Health Nursing, College of Nursing, King Saud University, Riyadh, Saudi Arabia; 6 Monash Nursing and Midwifery, Monash University, Melbourne, Victoria, Australia; College of Nursing, Qassim University, SAUDI ARABIA

## Abstract

**Background:**

Poor sleep quality during pregnancy has been increasingly associated with adverse neonatal outcomes and heightened maternal psychological distress. However, evidence from Middle Eastern populations remains limited, particularly in Saudi Arabia where cultural and environmental factors may influence sleep during pregnancy.

**Aim:**

To examine the association between maternal sleep quality during pregnancy and adverse neonatal outcomes (ANO), including preterm birth, low Apgar scores, and neonatal intensive care unit (NICU) admission, among postpartum women in Riyadh, Saudi Arabia.

**Methods:**

This cross-sectional study recruited 223 postpartum women via online platforms; 145 met inclusion criteria and were included in the final analysis. Sleep quality was assessed using a culturally adapted 10-item Arabic Maternal Sleep Quality (MSQ) scale, a culturally adapted 10-item Arabic Maternal Sleep Quality (MSQ) scale that underwent expert review and demonstrated strong internal consistency (Cronbach’s α = 0.88). Maternal psychological well-being and neonatal outcomes were self-reported. Multivariate logistic regression was conducted to identify predictors of ANO, adjusting for confounders.

**Results:**

Approximately 38.1% of women reported poor sleep quality, and 18.0% experienced at least one adverse neonatal outcome. Poor maternal sleep quality was associated with higher odds of ANO (adjusted OR = 1.75, 95% CI: 1.18–2.59, p = 0.005). A strong positive correlation was found between poor sleep and postpartum psychological distress (r = 0.52, p < .001).

**Conclusions:**

Poor maternal sleep quality is significantly associated with adverse neonatal outcomes and increased maternal psychological burden. These findings support consideration of routine sleep screening and culturally sensitive sleep-health counseling within antenatal nursing care in Saudi Arabia.

## Introduction

Pregnancy involves profound physiological, psychological, and social changes, during which maternal well-being directly affects fetal development [1]. Among factors influencing perinatal health, maternal sleep quality is increasingly recognized as critical yet often overlooked. Physiological changes, such as hormonal fluctuations, increased metabolic demands, and physical discomfort, frequently disrupt sleep, leading to longer sleep onset latency, nocturnal awakenings, reduced total sleep time, and lower sleep efficiency [2]. While often considered a normal discomfort of pregnancy, poor sleep has been linked to clinically significant maternal and neonatal complications [3].

International research consistently shows that inadequate or disrupted maternal sleep is linked to adverse obstetric outcomes, including gestational hypertension, preeclampsia, gestational diabetes, prolonged labor, and higher cesarean rates, as well as preterm birth and neonatal complications [4,5]. These effects are mediated by multiple biological pathways: chronic sleep deprivation can trigger systemic inflammation and oxidative stress, leading to placental dysfunction, while dysregulation of the hypothalamic-pituitary-adrenal (HPA) axis elevates maternal cortisol, disrupts glucose metabolism, and exacerbates hypertensive disorders complications [6,7]. Together, these mechanisms underscore the critical role of maternal sleep in supporting maternal and fetal health.

Beyond physiological outcomes, maternal sleep quality has important psychological implications. Insomnia and fragmented sleep during pregnancy are associated with increased peripartum anxiety, depressive symptoms, and stress [8].These psychological burdens can impair maternal well-being, hinder bonding with the newborn, reduce breastfeeding success, and challenge early parenting adaptation [9]. The bidirectional relationship between sleep and mental health highlights the need for interventions addressing both dimensions, with nurses, midwives, and maternal health providers playing a key role in early identification and supportive management [10].

Despite global recognition of sleep as a key determinant of perinatal health, region-specific knowledge gaps persist. In Saudi Arabia and other Middle Eastern countries, cultural, social, and environmental factors, such as evening social activities, multigenerational households, late bedtimes, and urban stressors like light pollution, traffic, and occupational demands, may uniquely influence maternal sleep patterns [11]. Yet, empirical studies examining how these factors affect maternal sleep and neonatal outcomes in Saudi Arabia remain limited [11].

The need for population-specific evidence is highlighted by the limited integration of sleep health into routine antenatal care in the region [12]. Current programs in Saudi Arabia focus on nutrition, blood pressure, diabetes screening, and fetal growth monitoring [13], while sleep assessment is rarely incorporated despite its impact on maternal and neonatal outcomes. From a nursing perspective, this gap represents both a challenge and an opportunity [14]; as frontline providers, nurses have frequent contact with pregnant women and are well-positioned to promote sleep health, identify risks, and provide culturally appropriate counseling. Incorporating simple sleep screening tools into antenatal care could offer a cost-effective, scalable intervention [15,16].

Another pressing consideration is the paucity of validated Arabic-language tools for assessing maternal sleep quality. Most existing instruments, such as the Pittsburgh Sleep Quality Index, were developed and validated in Western contexts [17]. While these tools provide valuable frameworks, cultural adaptation and psychometric testing are required to ensure their reliability and validity among Saudi women. Without culturally adapted instruments, healthcare providers may underestimate the prevalence of sleep disturbances or fail to capture their nuanced manifestations. Developing and validating such tools is therefore an essential step toward building evidence-based nursing practice in this domain.

Overall, there remains limited evidence on the relationship between maternal sleep quality and neonatal outcomes within the Saudi cultural and healthcare context, particularly from a nursing perspective. Addressing this gap will inform culturally sensitive antenatal interventions and strengthen maternal–child health practice, policy, and research in Saudi Arabia.

### Aim of the study

The primary aim of this study was to examine the association between maternal sleep quality during pregnancy and adverse neonatal outcomes, including preterm birth, NICU admission, and low Apgar scores, among postpartum women in Riyadh, Saudi Arabia. Secondary aims were to validate a culturally adapted maternal sleep quality scale, estimate the prevalence of poor sleep and adverse neonatal outcomes, and explore the relationship between maternal sleep quality and postpartum psychological well-being.

### Research question

- **Primary research question**

What is the association between maternal sleep quality during pregnancy and adverse neonatal outcomes (preterm birth, NICU admission, and low Apgar scores) among postpartum women in Riyadh, Saudi Arabia?

- **Secondary research questions**
  1. What is the prevalence of poor maternal sleep quality and adverse neonatal outcomes in this population?
  2. How reliable is the Arabic version of the maternal sleep quality scale?
  3. What is the relationship between maternal sleep quality and maternal psychological well-being in the postpartum period?

## Methods

### Study design and setting

This study employed a cross-sectional design and was conducted in Riyadh, Saudi Arabia, between January and March 2025. The design adhered to the STROBE (Strengthening the Reporting of Observational Studies in Epidemiology) guidelines to ensure methodological rigor and transparency. Riyadh, as the capital and most populous city in the Kingdom of Saudi Arabia, provided a culturally diverse urban population suitable for assessing maternal health indicators. The focus on postpartum women allowed retrospective assessment of maternal sleep during pregnancy and its relationship with immediate neonatal outcomes.

### Sample and sampling

The study targeted postpartum women residing in Riyadh who had delivered within the previous six weeks. Participants were recruited between 1 January 2025 and 31 March 2025. Inclusion criteria were: (1) current residency in Riyadh, (2) ability to read Arabic and provide informed consent, and (3) completion of the study questionnaire. Women were excluded if they had multiple gestation pregnancies or severe medical conditions unrelated to sleep but known to significantly affect neonatal outcomes (e.g., fetal anomalies diagnosed prenatally). Informed consent was obtained from all participants. Because data were collected via an online questionnaire, electronic informed consent was obtained, whereby participants reviewed an information page describing the study objectives, voluntary participation, anonymity, and data confidentiality, and proceeded to the survey only after indicating their consent. No minors were included in the study. Completion of the online questionnaire was considered evidence of electronic informed consent, as approved by the Institutional Review Board.

A non-probability sampling approach was used, combining online dissemination, snowball sampling, and recruitment through professional nursing networks and hospital maternity units. In total, 223 women initiated the survey. Of these, 78 were excluded for the following reasons: multiple gestation (n = 12), presence of severe medical conditions known to affect neonatal outcomes (n = 15), residence outside Riyadh (n = 18), delivery more than six weeks prior to survey completion (n = 11), duplicate entries (n = 7), and incomplete responses that did not meet the analysis requirements (n = 15). The final analyzed sample comprised 145 participants.

### Data collection tools

The primary instrument was a structured Arabic-language questionnaire developed specifically for this study to ensure cultural and linguistic appropriateness. It comprised four sections: demographic characteristics, maternal sleep quality, neonatal outcomes, and maternal psychological well-being.

### Maternal Sleep Quality (MSQ) Scale

This 10-item scale was designed by the research team based on literature synthesis and adapted to the local cultural context. It aimed to comprehensively assess sleep dimensions relevant to pregnant women in Saudi Arabia. Items included: sleep onset latency, frequency of nocturnal awakenings, sufficiency of sleep (≥7 hours), morning refreshment, daytime sleepiness, pain-related sleep interference, psychological stress at bedtime, use of sleep aids (medications or relaxation techniques), presence of nightmares, and perceived overall impact on quality of life. All items were rated on a 5-point Likert scale (e.g., from “Never” to “Always”). Positively worded items were reverse-coded, and a composite score (mean of all items) was calculated. Higher scores indicated poorer sleep quality. The scale demonstrated high internal consistency (Cronbach’s α = 0.88) and underwent forward-backward Arabic translation by bilingual experts. Content validity was reviewed by three academic nursing faculty members and two clinical obstetric nurses. Pilot testing with 12 postpartum women ensured clarity and comprehension.

### Neonatal Outcomes Index

This section collected clinical information including gestational age at birth, birth weight, NICU admission status, Apgar scores at 1 and 5 minutes, feeding regularity on day one, early infant sleep, respiratory complications, and postnatal immunization response. The primary outcome was a composite Adverse Neonatal Outcome (ANO), defined as the presence of any of the following: preterm birth (<37 weeks), NICU admission, or Apgar score <7 at 1 or 5 minutes. All neonatal outcomes were self-reported by participants and were not verified against medical records.

### Maternal Psychological Burden Scale

A 6-item measure developed by the research team assessed emotional bonding, psychological strain, coping under sleep deprivation, and perceived support. Items used a 5-point Likert scale, and higher scores indicated greater distress. This scale also demonstrated acceptable reliability (Cronbach’s α = 0.82) and was culturally adapted through expert review and pilot testing.

### Data collection procedure

Data collection was carried out using a structured online questionnaire that had been developed in Arabic to ensure cultural and linguistic appropriateness. Recruitment announcements were circulated through online platforms, maternity social media groups, and professional nursing networks in Riyadh. Eligible participants who met the inclusion criteria were provided with a secure link to the electronic questionnaire. The online format allowed women to complete the survey at their convenience within the first six weeks postpartum. This approach minimized logistical barriers, expanded the geographical reach within Riyadh, and ensured participant privacy. To enhance data integrity, the online survey was designed to prevent incomplete submissions by requiring responses to all essential items before progression. Completion of the online questionnaire was considered as implied informed consent, consistent with the approved ethical protocol.

### Data analysis

Data were analyzed using IBM SPSS Version 28. Descriptive statistics summarized demographics, sleep quality, and neonatal outcomes. Internal consistency of multi-item scales was evaluated using Cronbach’s alpha. Correlational analyses (Pearson’s r) explored associations between maternal sleep quality and psychological burden. For the primary analysis, multivariable logistic regression was used to assess the association between the MSQ score (continuous) and the ANO composite, adjusting for maternal age, parity, and history of sleep disorders. Model fit was assessed using the Hosmer–Lemeshow goodness-of-fit test, and classification accuracy was calculated.

Missing data were minimized by configuring the online questionnaire to prevent incomplete submission of essential items. Data were screened prior to analysis; analyses were conducted on complete cases for the variables included in each model.

### Ethical considerations

This study was approved by the Institutional Review Board of King Saud University, Riyadh, Saudi Arabia (IRB approval number KSU-HE #24-963). Participants were recruited between January 1, 2025 and March 31, 2025.

All participants provided electronic informed consent prior to participation. An online information page explained the study purpose, procedures, voluntary nature of participation, anonymity, and data confidentiality. Participants were required to confirm consent before accessing the questionnaire. No identifiable personal information was collected, and researchers did not have access to information that could identify individual participants during or after data collection. The study included adult postpartum women only; no minors were enrolled. All procedures were conducted in accordance with the ethical standards of the institutional research committee and the principles of the Declaration of Helsinki.

## Results

### Participant characteristics

The study included 145 postpartum women from Riyadh, Saudi Arabia. The mean age of the participants was 30.8 years (standard deviation [SD] = 5.1). The age distribution was as follows: 14.5% were 20–25 years old, 31.7% were 26–30 years, 30.3% were 31–35 years, 18.6% were 36–40 years, and 4.8% were ≥41 years. Nearly half of the participants (47.6%) were multiparous, defined as having three or more previous pregnancies. The majority of deliveries (74.5%) occurred at term (37–40 weeks gestational age). A self-reported history of a prior sleep disorder was present in 18.6% of the participants. These demographic characteristics are summarized in Table 1.

**Table 1:** Participant characteristics (n = 145).

Characteristic	Category	n (%)
**Age**	20–25	21 (14.5)
	26–30	46 (31.7)
	31–35	44 (30.3)
	36–40	27 (18.6)
	≥41	7 (4.8)
**Parity (prior pregnancies)**	1	32 (22.1)
	2	44 (30.3)
	3	36 (24.8)
	4	18 (12.4)
	≥5	15 (10.3)
**Gestational age at delivery**	<28 weeks	1 (0.7)
	28–31 weeks	3 (2.1)
	32–36 weeks	14 (9.7)
	37–40 weeks	108 (74.5)
	>40 weeks	19 (13.1)
**Prior sleep disorder**	Yes	27 (18.6)
	No	118 (81.4)

### Scale reliability

Internal consistency reliability for the multi-item scales was assessed using Cronbach’s alpha, as presented in Table 2. The Maternal Sleep Quality (MSQ) scale, comprising 10 items, demonstrated excellent internal consistency with a Cronbach’s alpha of 0.88. Similarly, the Neonatal Outcomes Index (an 8-item secondary composite) showed good reliability (α = 0.81).

**Table 2:** Reliability of study scales.

Scale	Items	Cronbach’s α
Maternal Sleep Quality (MSQ)	10	0.88
Neonatal outcomes index (secondary composite)	8	0.81
Maternal mental-health/relationship	6	0.90

### Maternal sleep quality levels

The mean composite score for Maternal Sleep Quality (MSQ) was 2.94 (SD = 0.67) on a 1–5 scale, where higher scores indicate poorer sleep quality. Detailed item-level analysis of the MSQ scale revealed significant sleep disturbances among the participants (Table 3). Specifically, 62% of mothers reported experiencing frequent nighttime awakenings, and 48% reported frequent daytime sleepiness. Pain was identified as interfering with sleep by 37% of the participants. Approximately 19% of the women reported using sleep aids “sometimes” or more frequently. Only 29% of participants reported feeling refreshed upon waking, indicating a widespread perception of insufficient restorative sleep.

**Table 3:** Maternal sleep quality (MSQ) descriptive statistics.

Measure	Value
MSQ mean (SD), range 1–5; higher = worse	2.94 (0.67)
Night awakenings often/always	62%
Daytime sleepiness often/always	48%
Pain interfering with sleep often/always	37%
Uses sleep aids ≥ “sometimes”	19%
Morning refreshed often/always (reverse-coded)	29%

### Neonatal outcomes

The prevalence of key neonatal outcomes is detailed in Table 4. Preterm birth (defined as <37 weeks gestational age) occurred in 12.4% of the cases. The majority of neonates (83.4%) had a birthweight within the normal range (2500–4000 g), while low birthweight (<2500 g) and high birthweight (>4000 g) each accounted for 8.3% of the births. Admission to the Neonatal Intensive Care Unit (NICU) was required for 10.3% of the neonates. An Apgar score of less than 7 at either 1 or 5 minutes was recorded for 8.3% of the newborns. The primary composite outcome, Adverse Neonatal Outcome (ANO), which includes preterm birth, NICU admission, or an Apgar score <7, was observed in 18.0% of the study population.

**Table 4:** Neonatal outcomes.

Outcome	n (%)
Preterm birth <37 weeks	18 (12.4)
Birthweight 2500–4000 g	121 (83.4)
Low birthweight <2500 g	12 (8.3)
High birthweight >4000 g	12 (8.3)
NICU admission	15 (10.3)
Apgar <7 at 1 and/or 5 min	12 (8.3)
Feeding regular day 1	104 (71.7)
Early infant sleep healthy	99 (68.3)
No respiratory distress	125 (86.2)
No vaccine complications	141 (97.2)
Composite ANO	26 (18.0)

### Bivariate associations

Bivariate analyses revealed significant associations between maternal sleep quality and other study variables, as summarized in Table 5. A strong positive correlation was found between the Maternal Sleep Quality (MSQ) score and the maternal psychological burden/relationship scale (r = 0.52, p < 0.001). This indicates that poorer antenatal sleep quality was significantly associated with a higher postpartum psychological burden and perceived strain in mother–infant bonding. A significant positive correlation also existed between the MSQ score and the neonatal outcomes index (r = 0.28, p = 0.001), suggesting that worse antenatal sleep was associated with a greater number of adverse neonatal indicators. Furthermore, when examining the prevalence of ANO across MSQ tertiles, a clear dose–response pattern was observed: the ANO prevalence was 9.8% in the best sleep tertile, 16.3% in the middle tertile, and 27.5% in the worst sleep tertile (Chi-square = 6.82, p = 0.033).

**Table 5:** Correlations among scales.

Pair	r	p-value
MSQ vs maternal psychological scale	0.52	<0.001
MSQ vs neonatal outcomes index	0.28	0.001
Maternal psychological vs neonatal outcomes index	0.23	0.006

### Multivariable logistic regression predicting ANO

Multivariable logistic regression was performed to predict the composite Adverse Neonatal Outcome (ANO), with the results presented in Table 6. After controlling for potential confounders including maternal age, parity, and self-reported prior sleep disorder, each one-point worsening in the MSQ score was associated with significantly higher odds of ANO (Odds Ratio [OR] = 1.75, 95% Confidence Interval [CI] 1.18–2.59, p = 0.005). A prior sleep disorder was also identified as a significant predictor of ANO (OR = 1.88, 95% CI 1.01–3.52, p = 0.047). Maternal age and parity were not significant predictors in the adjusted model. The overall model fit was acceptable, as indicated by a Hosmer–Lemeshow p-value of 0.41 and a classification accuracy of 77%.

**Table 6:** Logistic regression predicting ANO (n = 145).

Predictor	OR	95% CI	p-value
MSQ (per 1-point worse)	1.75	1.18–2.59	0.005
Age (years)	1.03	0.97–1.09	0.29
Parity (count)	0.94	0.80–1.10	0.44
Prior sleep disorder (yes vs no)	1.88	1.01–3.52	0.047
**Model fit**	Hosmer–Lemeshow p = 0.41	Classification accuracy = 77%	

Notes: ANO = composite of preterm birth, NICU admission, or Apgar <7 at 1 or 5 minutes.

## Discussion

This study explored the association between maternal sleep quality during pregnancy and a composite of adverse neonatal outcomes (ANO); including preterm birth, NICU admission, and low Apgar scores; in a sample of postpartum women in Riyadh, Saudi Arabia. Our findings demonstrate a statistically and clinically significant relationship: poorer maternal sleep quality, measured via a culturally adapted 10-item Arabic Maternal Sleep Quality (MSQ) scale with strong internal consistency, was associated with a 75% increase in the odds of experiencing an ANO. Additionally, poor sleep quality correlated with elevated maternal psychological burden in the early postpartum period. These findings align with global research trends and provide regionally specific evidence to inform nursing-led perinatal care strategies.

Several biological and psychosocial mechanisms support the observed link between poor sleep quality and adverse birth outcomes. During pregnancy, insufficient or fragmented sleep has been shown to provoke systemic inflammation, increase oxidative stress, and dysregulate the hypothalamic–pituitary–adrenal (HPA) axis, which can impair placental function and elevate risks of preterm labor and fetal compromise [18,19]. Specifically, elevated cortisol levels resulting from chronic sleep disruption may induce uterine contractility or vascular dysfunction, contributing to earlier deliveries or reduced fetal growth [20]. Moreover, poor sleep negatively affects glucose regulation, which can increase susceptibility to gestational diabetes—another independent risk factor for ANO [21].

The current study’s finding that maternal psychological distress is strongly correlated with poor antenatal sleep quality (r = 0.52, p < 0.001) is consistent with established evidence highlighting the bidirectional relationship between sleep and mood disorders [22]. Insomnia and poor sleep during pregnancy are known predictors of perinatal depression and anxiety, which in turn may hinder maternal–infant bonding, breastfeeding success, and responsive caregiving during the neonatal period [23]. The psychological stress experienced during pregnancy may further potentiate physiological stress responses, compounding risks for both mother and infant [24].

These findings are also consistent with the broader literature concerning maternal sleep and neonatal health. A study concluded that poor sleep quality during pregnancy is associated with higher odds of preterm birth, low birthweight, and NICU admission [25]. A similar review found that short sleep duration and poor sleep efficiency were linked to adverse birth outcomes, with pooled effect sizes comparable to those observed in the current study [26]. study also demonstrated that sleep-disordered breathing in pregnancy increases the risk of hypertensive disorders and cesarean delivery [27]. Our use of a composite ANO metric reflects current trends toward multidimensional neonatal outcome measurement, increasing the ecological validity and clinical relevance of the findings [28].

The mean MSQ score of 2.94 (SD 0.67) in our sample aligns with findings from similar populations, where third-trimester sleep disturbances peak due to increased physical discomfort, nocturia, and hormonal changes [29]. Specifically, 62% of participants reported frequent nocturnal awakenings, while 48% experienced daytime sleepiness—figures nearly identical to global estimates [30]. Only 29% reported feeling refreshed upon waking, highlighting the widespread burden of nonrestorative sleep among pregnant women in urban Saudi settings.

Of particular interest is the dose–response pattern observed in the prevalence of ANO across MSQ tertiles: ANO rates increased from 9.8% in the best sleep group to 27.5% in the worst. This trend suggests a dose–response association, whereby poorer sleep quality was observed alongside higher ANO prevalence; however, causality cannot be inferred from this cross-sectional design. This pattern has also been observed in prior studies, who reported that each hour decrease in maternal sleep duration increased preterm birth risk by 25%.

Importantly, this study contributes to the limited body of research on maternal sleep in the Middle East. Cultural norms in Saudi Arabia; including late-night family routines, high caffeine consumption, and reduced physical activity, may exacerbate sleep disruptions during pregnancy [31]. While these lifestyle factors were not directly assessed in this study, their potential influence underscores the importance of culturally sensitive sleep health promotion.

From a nursing perspective, the findings support the systematic inclusion of sleep assessment in routine antenatal care. Nurses are often the first and most frequent point of contact for pregnant women and are therefore uniquely positioned to identify and intervene in sleep-related issues. Routine use of brief screeners, such as the MSQ or adapted two-item versions, could facilitate early detection of sleep disturbances. Women identified at risk can benefit from nurse-led interventions, including education on sleep hygiene, positional therapy, and relaxation techniques [32,33].

Culturally appropriate sleep hygiene counseling is especially critical. Riyadh-based interventions should address common barriers to healthy sleep, such as evening screen use, heavy late meals, and environmental light exposure. Interventions can be delivered during clinic visits or through digital platforms, particularly given the demonstrated feasibility of online data collection in this study. Previous research has shown that Arabic-language digital tools can effectively improve maternal self-care and health literacy [34].

Further, nurses should be trained to recognize symptoms suggestive of sleep-disordered breathing or clinically significant insomnia and refer women to appropriate specialists. Integrating mental health screening, particularly for anxiety and depression, into antenatal care pathways is essential given the close linkage between psychological health and sleep quality.

### Implications for nursing practice

The findings of this study highlight the critical role nurses can play in mitigating adverse neonatal outcomes by addressing maternal sleep quality during pregnancy. Given that each one-point worsening in sleep quality increased the odds of an adverse neonatal outcome by 75%, incorporating routine sleep assessment into antenatal care is both justified and necessary. Nurses, as frontline providers in prenatal settings, are well-positioned to screen for sleep disturbances using brief validated tools, such as the MSQ scale or a 2–3 item screener assessing sleep latency, nighttime awakenings, and perceived restfulness.

Culturally adapted sleep hygiene counseling should be integrated into prenatal education programs. Topics should include the impact of late-night routines, excessive caffeine intake, electronic device usage before bed, and irregular sleep schedules; factors prevalent in urban Saudi lifestyles. Nurses can offer evidence-based behavioral interventions such as relaxation techniques, positional sleep aids, and pain relief strategies to improve sleep quality. Moreover, patients exhibiting signs of sleep-disordered breathing, severe insomnia, or comorbid mood disturbances should be referred to appropriate specialists, including sleep medicine or behavioral health providers.

Beyond pregnancy, postpartum education should emphasize sleep expectations and support strategies for new mothers, promoting continuity of care. Ultimately, a proactive, nurse-led approach to maternal sleep health may reduce NICU admissions, improve perinatal mental health, and enhance the quality of maternal-infant bonding.

### Strengths and limitations

This study offers several strengths that enhance its clinical relevance and methodological rigor. Foremost, the use of a culturally adapted, Arabic-language sleep quality scale with excellent internal consistency (Cronbach’s α = 0.88) ensures the tool’s contextual appropriateness for the Saudi population. Furthermore, the study focused on a composite adverse neonatal outcome (ANO), incorporating clinically meaningful indicators, preterm birth, NICU admission, and low Apgar scores, offering a robust metric to evaluate neonatal risk. The multivariable analysis accounted for key confounders such as maternal age, parity, and prior sleep disorder history, thereby improving model validity.

However, several limitations must be acknowledged. The cross-sectional design precludes causal inference, limiting conclusions about temporal relationships between sleep and neonatal outcomes. Data were collected via self-report questionnaires, which may introduce recall bias, particularly for postpartum reflections on antenatal sleep and for self-reported neonatal outcomes. Additionally, the study did not control for other potentially influential variables such as maternal BMI, socioeconomic status, comorbidities (e.g., hypertension, diabetes), work schedules, or environmental factors, all of which may influence both sleep quality and birth outcomes.

The sample size, while adequate for exploratory analysis, yielded only 26 ANO cases, limiting the power and precision of logistic regression models. A larger sample with a higher number of outcome events would provide more stable estimates and facilitate subgroup analyses. Lastly, the ANO composite, though useful for capturing overall risk, combines outcomes of differing etiologies and severities, potentially obscuring specific sleep–outcome associations.

### Future research

Future studies should employ longitudinal or prospective cohort designs to establish temporal and potentially causal relationships between maternal sleep quality and neonatal outcomes. Objective sleep measures, such as actigraphy, polysomnography, or wearable sleep monitors, should be incorporated alongside validated self-report tools to triangulate findings and reduce bias.

Moreover, future research should explore additional contextual factors influencing sleep in Middle Eastern populations, including work schedules (e.g., night shifts), household crowding, light and noise pollution, and dietary patterns. Research should also assess the interaction between sleep quality and modifiable risk factors such as physical activity, screen time, and mental health status.

Critically, randomized controlled trials (RCTs) evaluating nurse-led interventions, such as culturally tailored sleep hygiene education, mindfulness training, and cognitive-behavioral therapy for insomnia (CBT-I), are warranted to test their impact on both sleep outcomes and neonatal endpoints. Such trials should include implementation science frameworks to assess intervention feasibility, fidelity, and scalability within antenatal care systems across the Gulf region.

Finally, research should evaluate postpartum trajectories of maternal sleep and psychological recovery, particularly in relation to breastfeeding, infant sleep regulation, and family support. Such efforts will help shape integrated, lifespan-informed maternal–child health interventions rooted in nursing practice.

## Conclusions

This study provides compelling evidence that poor maternal sleep quality during pregnancy is significantly associated with increased odds of adverse neonatal outcomes and higher levels of maternal psychological distress. These findings are particularly salient within the Saudi context, where sleep disruptions during pregnancy may be shaped by cultural, environmental, and behavioral factors. The study underscores the urgent need to integrate comprehensive sleep screening and culturally informed counseling into routine antenatal nursing care.

By prioritizing maternal sleep health, nurses can contribute not only to reducing neonatal risks such as preterm birth and NICU admission but also to enhancing maternal well-being and early bonding experiences. These findings support a shift toward holistic, preventative, and nurse-led models of care that address sleep as a vital sign of perinatal health.
